# Development and Preliminary Validation of LoAD Calc, a Mobile App for Calculating the Maximum Safe Single Dose of Local Anesthetics

**DOI:** 10.3390/healthcare9070799

**Published:** 2021-06-25

**Authors:** Mélanie Suppan, Tal Sarah Beckmann, Cenan Gercekci, Thérèse Sigrist, Georges Louis Savoldelli, Roxane Fournier, Caroline Flora Samer

**Affiliations:** 1 Division of Anesthesiology, Department of Anesthesiology, Clinical Pharmacology, Intensive Care and Emergency Medicine, Geneva University Hospitals and Faculty of Medicine, 1211 Geneva, Switzerland; tal.beckmann@hcuge.ch (T.S.B.); georges.savoldelli@hcuge.ch (G.L.S.); roxane.fournier@hcuge.ch (R.F.); 2 Division of Clinical Pharmacology and Toxicology, Department of Anesthesiology, Clinical Pharmacology, Intensive Care and Emergency Medicine, Geneva University Hospitals and Faculty of Medicine, 1211 Geneva, Switzerland; cenan.gercekci@hcuge.ch (C.G.); caroline.samer@hcuge.ch (C.F.S.); 3 Pharmacy, Geneva University Hospitals, 1211 Geneva, Switzerland; therese.sigrist@hcuge.ch

**Keywords:** mobile apps, local anesthetics, toxicity, design thinking, development, dose calculation, information systems research, drug safety

## Abstract

Local anesthetics systemic toxicity can lead to life-threatening situations. Correct calculation of the maximum safe dose is therefore paramount in preventing such complications. Different solutions have already emerged to support anesthesiologists but are seldom used in clinical practice as they require either access to a computer or specific documents to be at hand. A mobile app could provide an easy and practical solution; however, the few apps already created for this purpose often lack key elements, allowing invalid data to be entered and suggesting doses that might exceed the maximum safe dose. We describe the development of LoAD Calc, a mobile health (mHealth) app developed using a modified version of the Information Systems Research framework, which adds design thinking modes to the original framework. The app was enhanced through multiple iterations and developed with the aid of contextual observations and interviews, brainswarming sessions, prototyping, and continuous feedback. The design process led to the creation of two prototypes which underwent thorough testing by a sample of eight anesthesiologists. The final version of the app, LoAD Calc, was deployed on Apple and Android mobile test platforms and tested again by the same sample until deemed fit for release.

## 1. Introduction

Local anesthetics (LAs) are widely used in medical practice, and anesthesiologists use them on a daily basis to perform locoregional anesthesia [1]. Local anesthetic systemic toxicity (LAST) is a rare but potentially life-threatening complication that can occur whenever these agents are used and are generally associated with central nervous system and cardiac toxicity [2]. In a recent summary, the incidence of LAST was reported to range from 0.04/1000 to 1.8/1000 [3]. However, an exact incidence is difficult to obtain as LAST symptoms and signs can be aspecific and evade prompt diagnosis even by experienced physicians [4]. Scientific societies have established recommendations for preventing LAST occurrence [3]. Among them, avoidance of both intravascular injection and administration of an inappropriately large dose is key to prevent toxic complications. Correct calculation of the maximum safe dose of LAs before administration is therefore paramount to decrease the occurrence of LAST.

Although basic rules for determining the maximum LA dosage might seem straightforward at first glance, an actual calculation can be difficult to achieve both safely and accurately in stressful clinical situations or when mixtures of different LAs are used. The maximum safe dose of LA can also be difficult to compute due to multiple confounding factors such as patient comorbidities and ideal body weight calculation [5].

Different solutions have already been developed to support physicians in LA dosage determination. The nomogram created by Williams in 2014 allows a rapid and calculation-free computation of the maximum safe dose of LAs [6]. The main limitation of this solution is that the nomogram must always be at hand. Moreover, specificities such as ideal body weight (IBW) calculation and adaptation in case of relevant comorbidities are indicated but not directly integrated into dose determination.

Computer-based solutions and mobile apps have been created but often lack key elements, allowing invalid data to be entered and therefore insufficiently focusing on safety issues. Most of them suggest doses that might exceed the maximum safe dose and give only general advice as to the caution with which their propositions should be used. The successful use of specific mobile health (mHealth) point of care decision-support tools focusing on drug administration has been described in other contexts, such as emergency settings [7], simulated pediatric cardiopulmonary resuscitation [8], as well as for the prescription of antibiotics [9].

The aim of this study was to develop and validate an mHealth app for the calculation of the maximum single safe dose of LAs, tailored to patients’ specifications and allowing the use of a mixture of two different LAs.

## 2. Materials and Methods

### 2.1. General Design

**Figure 1:**
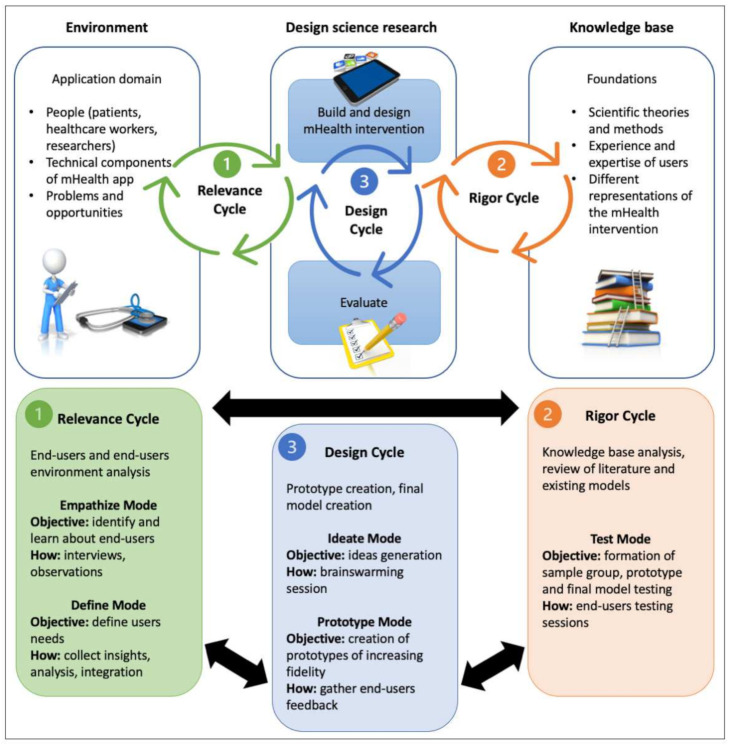
Modified Information Systems Research framework, incorporating modes of design thinking into the relevance, design, and rigor cycles. Adapted from Farao J. et al. “A user-centered Design framework for mHealth”, 2020 [10] (Creative Commons Attribution License).

A modified version of the Information Systems Research (ISR) framework (Figure 1) [10], which adds design thinking (DT) modes to the original framework [11,12], was used to define and describe the development process of this mobile app. DT is a non-linear, iterative process used to understand users, challenge assumptions, redefine problems and create innovative solutions to prototype and test. The five modes—Empathize, Define, Ideate, Prototype, and Test—are described here, one after the other, for the sake of clarity but must be considered to be intertwined, thereby allowing for continuous redefinition and adaptation of the whole process [13]. Figure 1

### 2.2. Relevance Cycle

The aim of the relevance cycle was to identify the different categories of end-users, and their needs regarding the determination of safe doses of LAs by using the “empathize” and “define” modes of DT.

#### 2.2.1. Empathize Mode

The target audience was defined by conducting semi-structured interviews with anesthesiologists (Table 1) and observing their current clinical practice in the Division of Anesthesiology of the Geneva University Hospitals, Geneva, Switzerland. Contextual observation and “on-field” interviews were used to identify the needs and expectations of end-users as to the creation of a tool for calculating maximum safe doses of LAs.

#### 2.2.2. Define Mode

The feedback from end-users and the clinical observations were collated, synthesized, and analyzed. This allowed the identification of features deemed necessary by the end-users and to assess the app’s technical requirements.

### 2.3. Rigor Cycle

The rigor cycle consisted of two phases. In the first phase, the existing literature regarding LA dosage calculation was reviewed. A search for already existing tools for computing maximum safe doses of LAs was conducted by MS on the Medline and Google search engines. The strengths and weaknesses of the tools found through this search were analyzed by MS and TSB. Both authors then identified, grouped, and selected relevant items linked to “ideal” LA dosage calculation. These items were then included in the first prototype of the mobile app. To make the calculation as safe and accurate as possible, clinical pharmacologists and toxicologists were included in the rigor cycle. Their role as experts was to validate the mathematical formulae and the assumptions made from sometimes scarce literature. They were also intended to help resolve any disagreement regarding the selection of items linked to LA dosage calculation.

#### Test Mode

In the second phase, a sample group of end-users was selected to test the app prototypes. The problems and comments reported during the test mode were recorded and addressed before moving to a more advanced prototype and, eventually, to the final app.

### 2.4. Design Cycle

The goal of the design cycle was to regroup all the data gathered through the previous cycles to generate new ideas in the ideate mode. This was used to create a vision for the design and creation of the final version of the app.

#### 2.4.1. Ideate Mode

Ideas were generated during a brainswarming session [14]. Brainswarming was preferred over brainstorming to ensure the involvement and input of every participant and to gather possible solutions in a timely manner. A brainswarming session was conducted by providing a whiteboard with the goal at the top and the available resources at the bottom. Each participant could then add ideas on sticky notes, thus progressively creating a graph evolving from both ends and shaping the creative process. Emerging ideas were discussed at the end of the session. The set goal was the development of an ideal mobile app to compute the maximum safe dose of LAs in the chosen setting.

#### 2.4.2. Prototype Mode

A low-fidelity prototype for calculation simulation was initially developed. This first prototype was tested for the functionality and accuracy of the chosen formulae. It was then improved according to comments from the sample group.

Next, the second prototype of higher fidelity was developed to evaluate usability on a mobile device. This second version underwent testing by the same sample group.

The final high-fidelity version, LoAD Calc (for Local Anesthetics Dose Calculator), was then coded and deployed on multiple test platforms. It was incrementally improved through further rounds of testing.

## 3. Results

### 3.1. Relevance Cycle

**Figure 2:**
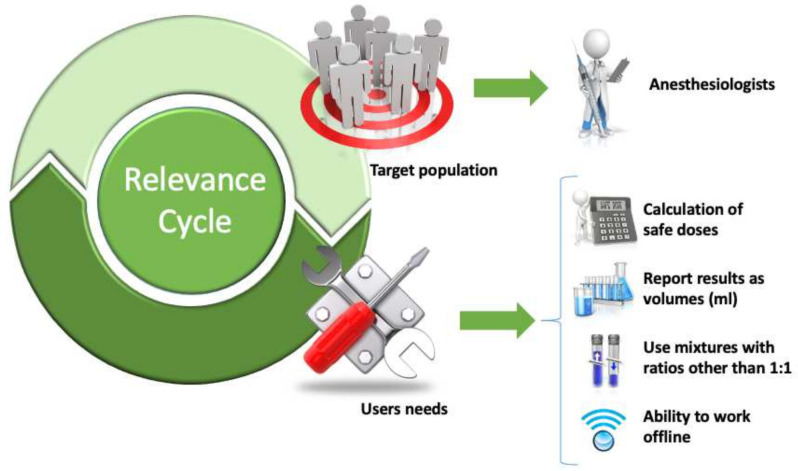
Results of the relevance cycle.

The results of the relevance cycle are summarized in Figure 2.

#### 3.1.1. Empathize Mode

Contextual interviews and observation of clinical practice led to the conclusion that many different healthcare professionals (anesthesiologists, certified nurse anesthetists, surgeons, emergency care physicians, etc.) use LAs on a regular basis and could benefit from the development of a dedicated mHealth app.

True to our initial aim, we decided to keep the focus on the anesthesiologist population as our target audience. Anesthesiologists are indeed more proficient in the use of LAs and should be more aware of potential shortcomings. In addition, they often need to use high doses of various LAs in their practice. The decision to target this specific population was taken to simplify the DT process, accelerate the development process and increase the reliability of the final mobile app.

#### 3.1.2. Define Mode

Contextual interviews and observations confirmed that LA dosage calculation could be unclear when patients had multiple comorbidities or when more than one LA was administered. This was particularly true when anesthesiologists had to use mixtures with ratios other than 1:1, as there was no definite rule or tool to help them accurately compute the maximum safe dose. Most of the interviewed anesthesiologists were convinced that an app addressing these issues would be useful in their daily practice and would increase patient safety. All of them were comfortable with using a mobile app on their personal or professional smartphone. One of the recurring demands was that the app should work offline as many operating rooms are located underground with sometimes difficult access to the cellular network and a slow internet connection.

Though maximum safe doses are calculated as milligrams of local anesthetics, all anesthesiologists agreed that the app should give the results as volumes. In their daily clinical practice, anesthesiologists performing locoregional anesthesia refer to LA volume for a given LA concentration rather than to LA dosage. Indeed, once a volume has been determined, the anesthesiologist can immediately prepare the solution without any need for further calculations. Displaying a maximum dose in milligrams only would mean that further calculations would be required to determine the maximum volume.

### 3.2. Rigor Cycle

The search for already existing tools used for LA dose calculation allowed the identification of four solutions, three of which were electronic (Table 2). None of these solutions met the previously defined requirements. Data regarding the elements used for ideal dose calculations were most often scarce. They were classified into three categories: “known”, “unclear”, or “unknown”, depending on the level of evidence for each of them (Table 3). A selection of items to be included in the mHealth app was then made based on reasonable feasibility without having to perform further studies on the subject. “Unknown” items were therefore removed from the project’s scope. Nearly all “unclear” items were included after further literature search and elaboration of a proposition on how to safely include these items in the final calculation.

When in doubt, the solution leading to the safest (lowest) values was always preferred. The only “unclear” item not included in the app was the evaluation of a time-dependent maximum dosage because it was considered out of scope for this initial development.

The choice of LAs to be included and their respective concentrations were made upon current local practice for peripheral nerve blockade as a convenience sample (see the “adaptation” column in Table 3). It was decided, as a first step, to focus on LA dose calculation for adult patients only and to exclusively allow the use of the metric system for height and weight entries.

#### Test Mode

**Figure 3:**
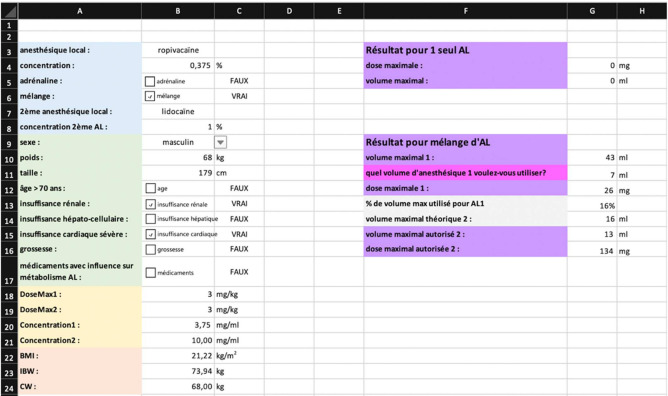
First (low fidelity) prototype developed under Microsoft Excel.

Eight anesthesiologists were recruited to test the low-fidelity prototype (Figure 3). All of them were able to use this prototype effectively, and their comments and suggestions for improvement were recorded. These issues were subsequently addressed, and a corrected low-fidelity prototype was created before moving on to a more advanced one.

### 3.3. Design Cycle

#### 3.3.1. Ideate Mode

The brainswarming session allowed the identification of many important concepts which helped avoid computational errors and improve usability, ergonomics, and safety. The concepts that were identified and used to build the app are summarized in Table 4.

Regarding the target platform, anesthesiologists found that it would be easier to use such an app on their mobile phones. Most of them use their own device at work (mainly iOS or Android) but also have access to a professional phone (Android). It was thus decided to create a multiplatform mobile app that could be used both on iOS and Android devices. To facilitate development, the coding language had to be similar for both platforms. After reviewing different possibilities, the Dart 2.9.0 programming language using the Flutter open-source development kit was selected for the development of the app. The widget-based coding architecture it uses and the comfort of the multiplatform deployment were decisive factors.

#### 3.3.2. Prototype Mode

The first low-fidelity prototype was created using macros in an Excel 2016 spreadsheet (Microsoft Corporation) (Figure 3). This version contained a first calculation simulation as well as all definitions and data. This allowed users to control each step of the calculation. The first round of testing uncovered some ineffective calculations or inaccuracies consecutive to logic errors in the formulas. This prototype was corrected and tested again until validation from the whole sample group was obtained.

**Figure 4:**
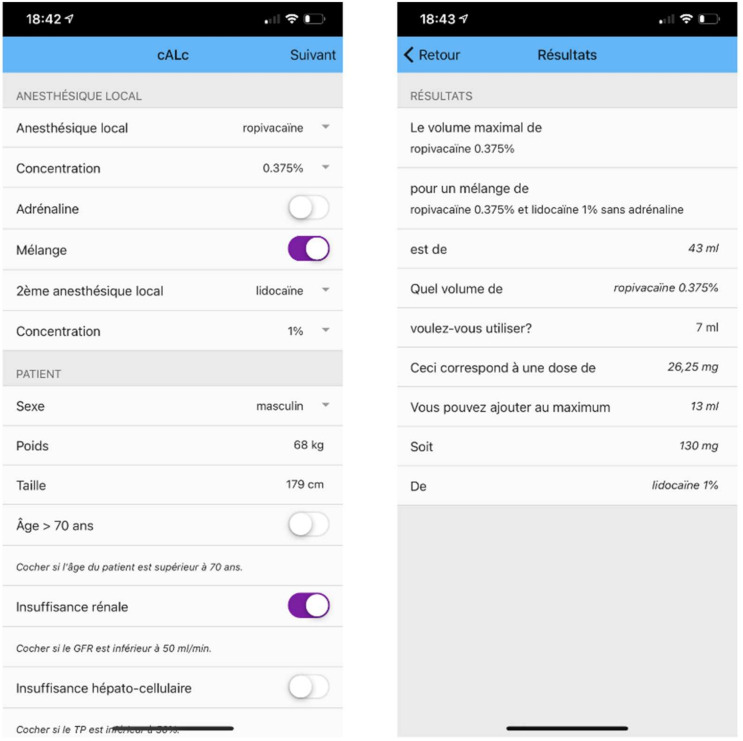
Second prototype developed with Calcapp (Neosupport AB).

A second prototype with improved fidelity was then developed with Calcapp (Neosupport AB) to test usability on a mobile device (Figure 4). Calcapp is a cloud-based app design engine that enables easy and rapid app development without programming. This second version underwent testing by the same sample group. At this stage, the main concerns raised were about ergonomic issues, such as misplaced buttons and the overall appearance of the app. As these issues could not be corrected on Calcapp itself, it was decided to move forward to the development of the final version of the app.

**Figure 5:**
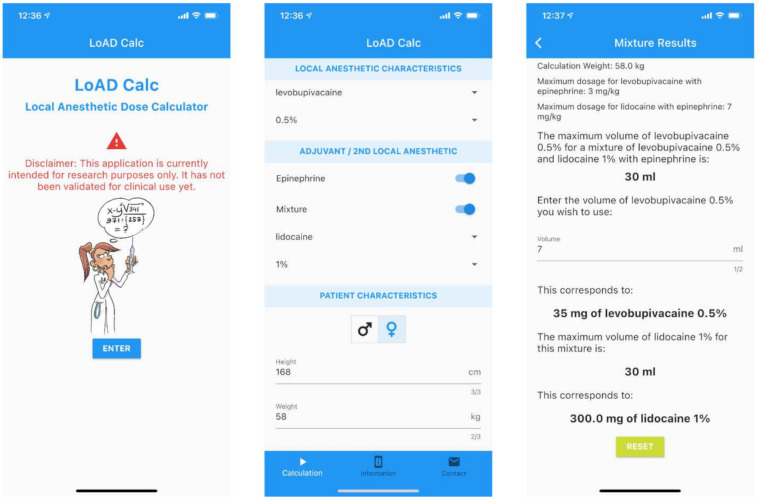
LoAD Calc, the final version of the app.

The final high-fidelity version, LoAD Calc, was coded in Dart 2.9.0 using the Flutter open-source development kit (Google, LLC, Mountain View, USA) and deployed for both the Apple Store (Testflight) and Google Play (Google Play Console) test platforms (Figure 5). The first two prototypes were developed in French as this was more convenient for the development team. However, the final version was entirely in English to enhance its adoption and generalizability. It was again tested by the sample group. Minor syntax and logic code errors, as well as ergonomic and usability issues, were corrected after the testing session (Table 5). An email sending form was included inside the app to allow easy reporting and feedback.

The iterative nature of this development prevents a precise and easily readable timeline from being provided. Two years had elapsed since the start of the LoAD Calc project to the release of the installation package.

## 4. Discussion

LoAD Calc, a mobile app designed to allow easy, fast and safe computation of maximum single doses of LAs, was successfully created using the modified ISR framework incorporating DT elements. The inclusion of end-users from the beginning of the project allowed identification of their needs and adaptation of the app consequently. Previous studies have demonstrated that mHealth developments are more prone to success when using a DT or User-Centered Design approach [27,28]. At the end of our intervention, the sample group of anesthesiologists was satisfied with the final version of the app and felt it was easy to operate and would be useful in their daily practice.

Mobile apps or other mHealth solutions are being developed for many different interventions in healthcare. There has been a recent and significant increase in the development of solutions aiming to reduce errors surrounding prescription and drug administration [7,8]. This kind of technological intervention is easy to target, rather fast to develop and can lead to significant improvements in clinical practice and patient safety. However, only a few solutions have been developed so far for LA dose calculation, and none are performing adequately in consideration of safety issues. Creating an app that would only propose safe doses was therefore identified as an appropriate development target.

Theoretical bases for LA dose calculation are often unclear, and specialized literature on the subject is scarce. Discrepancies in maximum recommended dosage also exist, thereby leading to multiplication of standards and, therefore, of risks [29]. Despite these difficulties, and as the main goal of this study was to improve safety surrounding LA administration, it was decided to always choose the solution leading to a lower and thus safer LA dose. Clinical pharmacologists and toxicologists were involved early in the process, and their expert opinion was sought to settle unclear issues and validate decisions and rules for calculation. Should other researchers be interested in testing and validating our calculation algorithm, Table 3 should provide all necessary information. The first author of this manuscript would be delighted to provide any additional information should the need arise.

LA administration should be less hazardous nowadays with modern anesthesiology practice. Methods to prevent toxicity include using small doses, performing incremental administrations, choosing LAs with less toxicity, and carrying out an aspiration test [30]. The increasing use of ultrasonography to guide peripheral nerve blocks decreases the risk of intravenous injection and improves precision, thereby decreasing the volumes of LAs required to produce nerve blockade [31,32]. While these practices, along with better knowledge and prevention of LAST [33], should decrease the risk of toxicity, other factors might balance this equation [34]. Indeed, time pressure is often present in the clinical context [35] and was identified as a source of error when the mental calculation of LA doses had to be performed [36]. Moreover, the increasing trend of locoregional and multimodal analgesia favors LA administration over long periods or by multiple routes in the perioperative phase and could also increase the risk of toxicity. In addition, LAs are used by many different specialists and, although their use and associated risks are well known by anesthesiologists, other physicians might be less aware of these specificities and thus jeopardize patient safety by misusing these drugs [37,38].

The development of LoAD Calc is in line with the actual trend towards personalized medicine [39]. The ability to compute a safe LA dose tailored to the patient’s specifications definitely increases safety when LAs must be administered. Time-dependent prediction when multiple doses are to be given, or determination of a coefficient for administration depending on specific sites and routes represent further areas of improvement of the current app. These developments will have to take into account particular situations, such as the conversion of epidural analgesia to epidural anesthesia. Indeed, epidural analgesia for labor-induced pain can last for hours and must sometimes be topped up by LAs with higher concentrations if an urgent cesarean section has to be performed. Finally, awareness and knowledge concerning LAs dose adaptation could also be improved through our mHealth app. To refine this aspect, details regarding dose adaptation according to specific parameters should be added in a future version.

Our study is not without limitations. First, although LoAD Calc was successfully developed, it was only tested by a limited sample group for usability and correctness of a calculation. Its actual impact and safety should now be tested and be compared to other means of LA dose computation, first by means of a simulation study, then in the actual clinical field. In Switzerland, apps used to calculate medication dosage are considered full-fledged medical devices [40]. We will therefore need to validate LoAD Calc as a medical device for clinical use. The next validation step will be carried out by presenting clinicians with specifically designed vignettes and assessing the accuracy and adequacy of LA dosage. After ascertaining the safety and efficiency of our app, obtaining federal approval, and establishing CE compliance, the current disclaimer will be removed, and large-scale deployment will follow.

However, such distribution cannot be considered before CE approval is obtained.

Another limitation is that the current app does not yet allow for a wide array of LA choices and can be used only for single-dose administration. Further developments will therefore be needed to compute safe doses for continuous or repeated LA administration.

It should also be acknowledged that the current version of the app is limited to adult patients and that variables can only be entered (and are only displayed) using the metric system. This could prevent it from being widely adopted in settings or regions different than ours. Nevertheless, these choices were made to enhance the ease and speed of development, and the framework is flexible enough to allow the development and deployment of many updates. Increasing the choice of LAs has already been identified as a necessary improvement, and other LAs will be added in future versions.

Furthermore, even though the use of smartphones and tablets is nowadays almost commonplace, using such a specific tool could be associated with an increased workload. Actual clinical studies should allow us to assess this increase, which might be reduced by allowing the app to automatically retrieve relevant patient data, such as weight or comorbidities. Alternatively, integrating the app, or at least its algorithms, into the anesthesiologic electronic health record (EHR) could limit the additional workload, but data security issues will have to be considered carefully. The current version of the app is devoid of such issues as no data which could allow patient identification is collected. Therefore, local EHR-related constraints will have to be taken into account, and we acknowledge that the current of the app is more a proof-of-concept than a definitive fixed solution.

Local EHR-related constraints will have to be taken into account, and we acknowledge that the current of the app is more a proof-of-concept than a definitive full-fledged solution.

Finally, some of the decisions and calculation rules that are used by the app are not backed by strong scientific evidence. Expert opinions were sought to make up for this gap of knowledge and to select the most logical and safest solutions. These calculation rules will have to be updated according to the results of new studies in the specific field of LA and of LAST.

## 5. Conclusions

The modified ISR framework led to the successful development of LoAD Calc, an mHealth app designed to allow easy, fast, and safe computation of the maximum single dose of LAs.

## Figures and Tables

**Table 1:** Questions asked during the semi-structured interviews.

Open-Ended Questions	Specific Questions
In what context do you use local anesthetics?	For what kind of procedures do you use LAs?
	Which LAs do you routinely use?
	Do you use LA mixtures? If yes, do you use ratios other than 1:1?
How do you determine LA^1^ dosage in your actual practice?	Are there specific rules or tools you know of?
	Do you use any of these rules or tools?
	Do you take patient comorbidities into account?
	According to you, are current rules and tools satisfactory? If no, what are the shortcomings you have identified? Is there a solution you would think about?
Do you carry a smartphone while at work?	Is it a personal or professional device?
	Are any medical apps installed on your device?
	If yes, are you comfortable using these apps?
	Do you think that a mobile app regarding LA dosage calculation could be useful in your daily practice? If yes, what features would you expect, and which of these features would you consider mandatory?
Is there anything else you would like to add?	

^1^ LA: Local Anesthetic.

**Table 2:** Existing tools for local anesthetic dose calculation.

Tool	Strengths	Weaknesses
Nomogram [6]	No need for calculation Users are instructed to use IBW ^1^ for calculation Limitation of body weight entry Limitation of volume for the mixture	Accessibility No dose adaptation based on health conditions and drug interactions
MDCalc Local Anesthetic Dosing Calculator (online/app) [15]	Accessibility Limitation of body weight entry	Use of actual body weight for calculation No warning is given if the calculated dose exceeds the maximum safe dose No mixture calculation No dose adaptation based on health conditions and drug interactions
The Podiatry Institute’s LA ^2^ Toxic Dose Calculator (Excel spreadsheet) [16]	Computer-based Volume for 1:1 mixture Maximum volumes indicated	Use of actual body weight for calculation Not easily usable on a smartphone No warning is given if the calculated dose exceeds the maximum safe dose No dose adaptation based on health conditions and drug interactions
SafeLocal mobile app by Johns Hopkins Digital [17]	Accessibility Mixture calculation Maximum dosage is adapted according to certain relevant comorbidities	Use of actual body weight for calculation No limitation of value entries (i.e., no maximum body weight defined) No warning is given if the calculated dose exceeds the maximum safe dose

^1^ IBW: Ideal Body Weight. ^2^ LA: Local Anesthetic.

**Table 3:** Determination of the rules needed to calculate the maximum safe dose of local anesthetics according to scientific evidence.

Dosage Element	Knowledge Classification	Commentary	Inclusion(Y ^1^/N ^2^)	App Rules
Dose limit for a single LA ^3^	Known [18,19,20]	Slight variations of maximum LA dosage. Choice of safer (lower) dosage.	Y	Levobupivacaine: 2 mg/kg (max 150 mg/dose) Lidocaine: 3 mg/kg (max 300 mg/dose) Ropivacaine: 3 mg/kg (max 225 mg/dose)
Influence of epinephrine on dose limit	Known [18,19,20]	Slight variations of maximum LA dosage in the presence of epinephrine. Choice of safer (lower) dosage.	Y	Levobupivacaine: 3 mg/kg (max 150 mg/dose) Lidocaine: 7 mg/kg (max 400 mg/dose) Ropivacaine: 3 mg/kg (max 225 mg/dose)
Influence of other adjuvants	Unknown	Some supposed effects, but unclear if maximum LA dose should be adapted.	N	None
Influence of injection site	Unknown [5,21]	Better and faster absorption if the injection site is well perfused, but no algorithm defined yet to adapt maximum LA dose calculation.	N	None
Determination of Calculation Weight	Unclear [6,22,23]	Exact formula and limits for LA dose calculation are not clearly defined.	Y	1. Calculation of BMI ^4^; 2. Calculation of IBW ^5^ (Devine Formula); 3. Application of the following algorithm to define CW ^6^: Weight ≤ 70 kg and BMI < 30 and IBW>weight → CW = weight; Weight ≤ 70 kg and BMI < 30 and IBW≤weight → CW = IBW; Weight ≤ 70 kg and BMI ≥ 30 → CW = IBW; Weight > 70 kg and IBW > 70 → CW = 70; Weight > 70 kg and IBW ≤ 70 → CW = IBW.
Dose adaptation depending on health conditions/drugs	Unclear [5,24]	Some uncomplete indications in the literature, especially after repeated administration.	Y	Conditions: Old age (>70 years); Renal dysfunction (GFR ^7^ < 50mL/min); Hepatic insufficiency (PT ^8^ < 50%); Heart failure (LVEF ^9^ ≤ 30%); Pregnancy; Drugs decreasing LA metabolism; List of drugs decreasing LA metabolism: Major CYP1A2 inhibitors: ciprofloxacin, norfloxacin, and fluvoxamine; Major CYP3A inhibitors: azole antifungals, macrolides, calcium channel blockers, HIV antiretroviral therapy, and tyrosine kinase inhibitors. If one condition is present, the calculator reduces the total maximum dose by 20%. If two or more conditions are present, the calculator reduces the total maximum dose by 30%.
Calculation rule for LA mixtures	Unclear [19,25]	General rule for calculation of LA mixtures. Unclear if there could be a synergistic effect.	Y	The app performs the following steps: 1. Calculation of maximum safe volume for first LA; 2. The user enters which volume of first LA is to be used (0–maximum volume); 3. Calculation of corresponding maximum dose of first LA and determination of the percentage of total maximum dose; 4. Calculation of maximum dose of second LA-based on remaining percentage of total maximum dose; 5. Calculation of maximum volume of second LA.
Time-dependent maximum dosage	Unclear [26]	Should be adaptable from known data, but too many parameters considered unknown or unclear to be included at this stage.	N	None

^1^ Y: Yes; ^2^ N: No; ^3^ LA: Local Anesthetic; ^4^ BMI: Body Mass Index; ^5^ IBW: Ideal Body Weight; ^6^ CW: Calculation Weight; ^7^ GFR: Glomerular Filtration Rate; ^8^ PT: Prothrombin Time; ^9^ LVEF: Left Ventricular Ejection Fraction.

**Table 4:** Concepts retained for app development.

Concept	Goal	Solution
Dependent dropdown menus for concentration selection	Force selection of correct concentration for a given LA ^1^. Avoid unusual mixtures and inattention errors.	Only usual concentrations for chosen LA shown in the dependent dropdown. Dependent dropdown resets if LA is changed.
Conditional appearance of 2nd set of dropdown menus	Avoid unnecessary information on the screen.	Second set of dropdowns not visible if mixture toggle button is “on”.
Limited selection of 2nd LA	Avoid performing an unnecessary mixture calculation. Avoid invalid data input.	Error message displayed when both selected LAs are identical, and calculation is impossible.
Mandatory unique sex selection	Force sex selection. Avoid error in IBW ^2^ calculation.	If sex is not selected, an error message stays on the screen and calculation is impossible Only one selection is possible (male or female)
Number-only character-limited input fields for height and weight	Avoid calculation error Avoid invalid data input.	Input field and contextual keyboard-only allow numbers to be entered. Input limitation of maximum three characters.
Value limitations for height and weight	Avoid invalid data input.	Minimum and maximum values setting for the input fields, if a value outside limits error message is displayed and calculation is impossible.
Scrolling screen	Facilitate main page design and navigation	User can scroll the whole screen to see all the fields.
Reset button	Facilitate voiding of the form for new calculation.	Reset button to void all fields Refocus on top of the form page on pressing.
Conditional calculation	Avoid calculation errors.	Calculation only possible when all fields are filled, and no error message is displayed.
Conditional navigation to results pages	Avoid specific unnecessary tasks Allow better adaptation of results page depending on type of calculation (simple or mixture).	Navigation to a different results page, depending on the state of the mixture toggle button
Conditional display of 2nd part of mixture results	Avoid unnecessary information on the screen.	If no volume of first LA is entered, the proposed volume of second LA is not displayed.
Value limitation for chosen volume of 1st LA	Avoid display of invalid data. Avert miscomprehension for the user.	Entered volume must be between 0 and maximum allowed volume (displayed on-screen); if not, an error message is displayed.
Rounding of clinically usable values	Avert miscomprehension for the user Increase safety.	All given volumes are floored to inferior integer.

^1^ LA: Local Anesthetic; ^2^ IBW: Ideal Body Weight.

**Table 5:** Corrections performed after testing the final version.

Problem	Explanation	Correction
Misplacement of the “Calculate” button	Users found it was more logical to have the “Reset” button on the left and the “Calculate” button on the right.	Exchange of buttons placement.
Keyboard remained visible after entering data in the number field	This issue was found unpractical by users as the keyboard covers part of the screen.	Ability to make the keyboard disappear by defocusing.
Data disappeared when pressing “back” button from the results page	Users found it would be more convenient to keep the data already entered when pressing the “Back” button on the results page as it allows to modify only chosen parameters.	Data in the form are still visible when pressing the “Back” button from the results page; If the “Reset” button is pressed on the results page, it brings the user back to a blank form.
Calculation weight and LA ^1^ maximum dosage (mg/kg) unknown	Users wanted these two parameters to be indicated so that they could better understand and redo the calculations.	Calculation weight and LA maximum dosage in mg/kg were made visible on the results pages.
Too much information under the “Drugs” checkbox	Users found that the whole list of drugs decreasing LA metabolism was too long and difficult to read. Moreover, this list could slightly differ depending on the chosen LA.	Information button icon added under the checkbox. On pressing, it shows a list of relevant drugs decreasing LA metabolism for the chosen LA.

^1^ LA: Local Anesthetic.

## Data Availability

Not applicable.
